# Preoperative and Postoperative Assessment of Ultrasonographic Measurement of Inferior Vena Cava: A Prospective, Observational Study

**DOI:** 10.3390/jcm7060145

**Published:** 2018-06-10

**Authors:** Ayhan Kaydu, Erhan Gokcek

**Affiliations:** Department of Anesthesiology, Diyarbakir Selahaddini Eyyubi State Hospital, Diyarbakir 21100, Turkey; gokcekerhan_44@hotmail.com

**Keywords:** ultrasonography, preoperative, postoperative, collapsibility index, inferior vena cava diameter

## Abstract

Background: Ultrasound measurement of dynamic changes in inferior vena cava (IVC) diameter and collapsibility index (CI) are used to estimate the fluid responsiveness and intravascular volume status. We conducted an analysis to quantify the sonographic measurement of IVC diameter changes in adult patients at the preoperative and postoperative periods. Methods: Ultrasonography was performed on 72 patients scheduled for surgery with American Society of Anesthesiologists physical status I to III. Quantitative assessments of the end-expiration (D_min_), end-inspiration (D_max_), and CI at preoperative and postoperative period were compared in a prospective, observational study. The patients received intravenous fluid according to standard protocol regimes peroperatively. Results: Ultrasonography of IVC measurement was unsuccessful in 12.5% of patients and 63 patients remained for analyses. The mean age was 43.29 ± 17.22 (range 18–86) years. The average diameter of the D_min_, D_max_, and dIVC preoperative and postoperative were 1.99 ± 0.31 vs. 2.05 ± 0.29 cm, 1.72 ± 0.33 vs. 1.74 ± 0.32 cm, 14.0 ± 9.60% vs. 15.14 ± 11.18%, respectively (*p* > 0.05). CI was positively associated preoperatively and postoperatively (regression coefficient = 0.438, *p* < 0.01). Conclusion: The diameter of the IVC did not change preoperatively and postoperatively in adult patients with standard fluid regimens. The parameters of the IVC diameter increased postoperatively according to the preoperative period.

## 1. Introduction

Perioperative fluid administration is an important issue that has been discussed for many years in anesthesiology practice [1]. The goal of the perioperative fluid management is to avoid acute renal failure, cardiac arrhythmias, inadequate tissue oxygenation, decreased blood flow to organ perfusion, hypotension due to hypovolemia, interstitial edema, and cardiopulmonary complications due to excess fluid [1,2]. Therefore, the management of the fluid status of the patient in the perioperative period is important in terms of postoperative mortality and morbidity [2].

The clinical findings, vital signs (blood pressure, heart rate), as well as hemodynamic parameters such as central venous pressure (CVP) and even pulmonary artery occlusion pressure (PAOP) have not been accurate in determining circulating blood volume during conventional perioperative fluid management [3]. Although systolic pressure and pulse pressure variations are successful methods for detecting fluid response, these do not improve patient outcome [4]. Despite improved patient outcome by esophageal Doppler-optimized fluid management, this method is not performed commonly for financial problems and practical reasons [5].

In recent years, ultrasonographic inferior vena cava (IVC) diameter measurement changes due to respiratory variations have become an important method to determine the fluid responsiveness [6]. Research has also demonstrated good correlation with right atrial cardiac functions and IVC diameter measurements [7]. The caval index of the IVC and the maximum diameter at the end of the expiration in spontaneous respiration were shown to be indicators of the fluid responsiveness in different clinical trials [8,9].

The aim of the present study was to determine the IVC diameter measurements with respiratory variability in spontaneous breathing patients who underwent surgical operations preoperatively and postoperatively with standard fluid regimens.

## 2. Material and Methods

### 2.1. Patients

This study was reviewed and approved by the institutional review board at the Diyarbakir Gazi Yasargil Training and Education Hospital (ID: 81, 2017). Written informed consent was obtained from all patients.

This prospective, observational research was performed in a single urban state hospital. The approval for the research was granted by the Institutional Ethics Committee (decision no 2017/81, Diyarbakir Training and Research Hospital Ethics Committee). Written and spoken informed consent was obtained from all patients. The inclusion criteria for the study were patients aged over 18 years with a body mass index (BMI) less than 40 kg/m^2^ and patients who understood the study protocol and informed consent. Patients with abnormal anatomy of the gastrointestinal tract (previous esophageal, hepatic, or gastric surgery, including hiatus hernia), pregnancy, a history of major peripheral vascular disease, increased intra-abdominal pressure, difficult airway problems, coronary artery disease, myocardial infarction in the past 3 months, stroke, congestive heart failure (with an ejection fraction less than 35%), severe chronic pulmonary diseases, renal dysfunction (creatinine > 2.2 mg/dL), abnormal coagulation values, or active abdominal skin infection were excluded from the study. Moreover, we excluded surgical types and comorbidities, which could cause excessive fluid changes between body compartments.

### 2.2. Preoperative Procedure

In the preoperative care unit, with the spontaneously breathing patients lying supine, gastric examination and ultrasonography were performed by an experienced practitioner (who had at least 5 months of gastric ultrasound experience and performed 50 IVC ultrasound examinations). The doctor who performed the ultrasonography did not affect anesthesia management and other processes of the patient. The procedure was performed by a 2–5 Mhz curvilinear array low frequency transducer (Sonosite^®^ M-Turbo, Bothell, WA, USA) and recorded digitally. The transducer was placed along the subcostal longitudinal axis. First, the right atrium entrance of the IVC was identified as two-dimensional. Pulse wave doppler was used to separate the IVC from aorta. The IVC diameter was measured in a 2-dimensional mode with an M-mode at 2–3 cm distal from the right atrium entrance. The IVC collapsibility index was calculated as (dIVC − CI) = (dIVC_max_ − dIVC_min_)/dIVC_max_ and defined as percentage (%).

### 2.3. Anesthesia Management

The 18 G or 20 G intravenous catheters were inserted to all patients. The noninvasive blood pressure (NIBP), standard electrocardiogram (ECG), SpO_2_ (peripheral oxygen saturation), and end tidal carbon dioxide were monitored preoperatively. The anesthetic induction was applied with midazolam (0.05–0.2 mg/kg) intravenously, fentanyl (1–2 mcg/kg) intravenously, propofol (2–2.5 mg/kg) intravenously, and rocuronium (0.6 mg/kg) intravenously. Anesthesia was maintained with 40–50% O_2_-air, MAC (minimum alveolar concentration) level inhalation gases for sevoflurane (1.45%) or desflurane (5.3%). The mechanical ventilation was adjusted as tidal volume (6–8 mL/kg), PaCO_2_ 35–40 mmHg, I:E ratio 1.2, VCV ventilation mode (Datex Ohmeda, S/5 Avance Healthcare, Helsinki, Finland) after orotracheal intubation. Anesthetic agents were adjusted to maintain heart rate <100 bpm and mean blood pressure (MAP) within 30% baseline. MAP >30% was treated with labetolol or gliserol trinitrate intravenously. Hypotension was treated firstly with fluid administration, and if not improved, then Ephedrine (5–15 mg) was applied intravenously. The bradycardia was an accepted heart rate less than at 45 bpm. If required, it was treated with atropine (0.015 mg/kg) intravenously. At the end of surgery, neuromuscular block was reversed with neostigmine (0.05 mg/kg) intravenously and atropine sulphate (0.015 mg/kg) intravenously.

Spinal anesthesia was applied in midline axis between the L2–3, L3–4, and L4–5 intervertebral space with the patients in the sitting position with 10–20 mg dosing of heavy bupivacaine according to surgery type. The atraumatic pencil point needles were used for neuroaxial anesthesia. The motor and sensory block level was evaluated by Bromage scale. The operation was allowed as motor and sensory block levels reached T4–T6 dermatome levels. The hemodynamic instabilities were treated with guidelines as described above.

The supraclavicular block was applied to the patient to be treated with peripheral neuroaxial block. After antisepsis of the block to be blocked, 2 mL of 2% arythmal infiltration was performed on the subcutaneous tissue. The supraclavicular approach used 22 G, 50 mm needle (Pajunk needle, Germany) for block applications; 20 mL 0.5% levobupivacaine + 10 mL 2% lidocaine solution was used as the local anesthetic mixture.

### 2.4. Intraoperative Fluid Management

The baseline fluid requirement in the perioperative period was calculated on the duration of fasting, the fluid shift toward third space, and the amount of bleeding. The basal fluid requirement was calculated for the first 10 kg weight 4 mL/h, 2 mL/h for the second 10 kg, and 1 mL/h for the rest. Fluid deficit in the preoperative period were calculated by the fasting time of the basal fluid requirement, and ½ of this amount was administered in the first hour of operation, ¼ in the second hour, and ¼ in the third hour. For intraoperative blood and insensible loss, 0–2 mL/kg fluid was infused for minimal surgical procedures (e.g., inguinal hernia repair), 2–4 mL/kg for moderate surgical procedures (e.g., cholecystectomy), and 4–8 mL/kg for severe surgical procedures.

### 2.5. Postperative Procedure

All patients were followed at the postanesthesia care unit (PACU) for at least 30 min. IVC ultrasonography was performed on spontaneously breathing patients lying supine after pain management with Tramadol (0.8–1 mg/kg) if required, by the same experienced physician. The doctor who performed the ultrasonography was not aware of anesthesia management and the other processes of the patient. The procedure was repeated as described above preoperatively. Patients who were suspected of increase in postoperative intra-abdominal pressure were not included in the USG procedure.

### 2.6. Data Collection

During the study period, the data of the patients were recorded prospectively. The age, gender, height, weight, body mass index (calculated according to BMI = weight/height^2^ formula), types of surgery, applied anesthesia techniques (general anesthesia, spinal anesthesia, peripheral nerve blocks), preoperative and postoperative IVC values (IVC diameter at inspiration and expiration), amount of peroperative fluid, and peroperative hemodynamic values were recorded.

### 2.7. Statistical Analyses

In this study, to demonstrate the results, a descriptive analysis of the demographic data (age, weight, height, and BMI), gender, and ASA classifications were used. The data were summarized using the mean and standard deviation. The Shapiro–Wilk test was used for the assumption of normal distribution of continuous variables. If variables were normally distributed, central tendency was expressed as the mean (SD). Means were compared using independent or paired Student’s *t*-test. Spearman correlation analysis was used to find out a correlation between non-normally distributed independent variables. The Fisher exact test was used for categorical data and expressed in count, percentages. Differences were considered significant if *p* < 0.05. Statistical analysis was performed using SPSS 22 (Chicago, IL, USA).

## 3. Results

**Figure 1:**
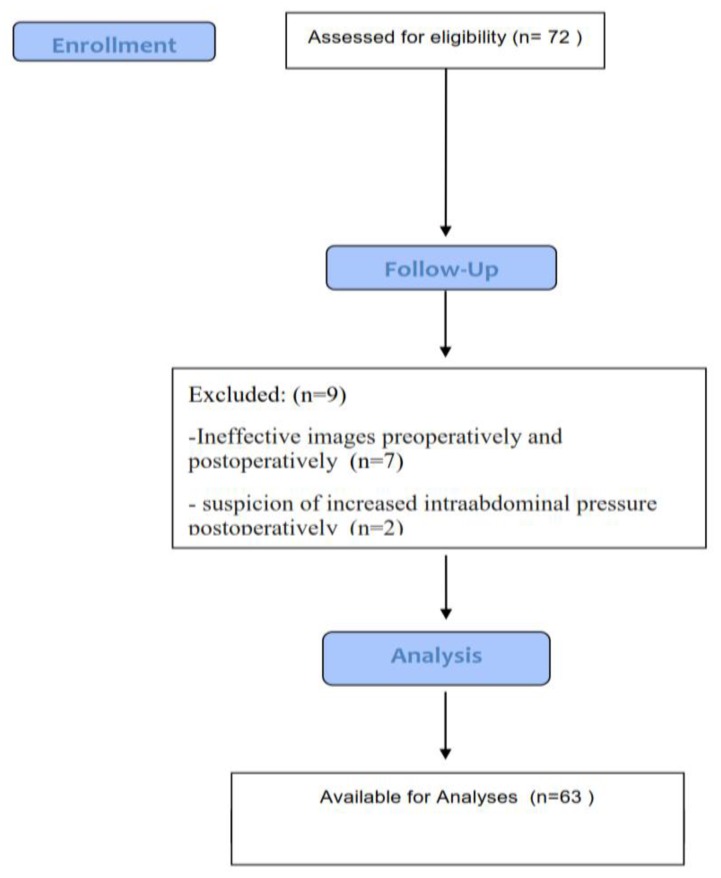
Flow chart.

Seventy-two patients were recruited in the study. Ultrasonography of IVC measurement was unsuccessful in 12.5% of patients. A total of 9 patients were excluded; in 7 patients detailed images could not be taken effectively preoperatively and postoperatively, and 2 patients had suspicion of intra-abdominal pressure increase postoperatively due to abdominal surgery. Therefore, evaluation was made with 63 patients (Figure 1). The patients comprised 32 males and 31 females with a mean age of 43.29 ± 17.22 years (range, 18–86 years). Average body mass index was 25.73 ± 4.07 kg/m^2^. The perioperatively mean infused fluid was 985.80 ± 484.27 mL. The demographic data of the patients are shown in Table 1. The following surgical operations were included: general (*n* = 33), orthopedic (*n* = 16), urology (*n* = 6), otolaryngology (*n* = 4), neurosurgical (*n* = 4). The comorbidities were six patients with hypertension (*n* = 6), diabetes mellitus (*n* = 4), cardiovascular diseases and respiratory (*n* = 2).

**Figure 2:**
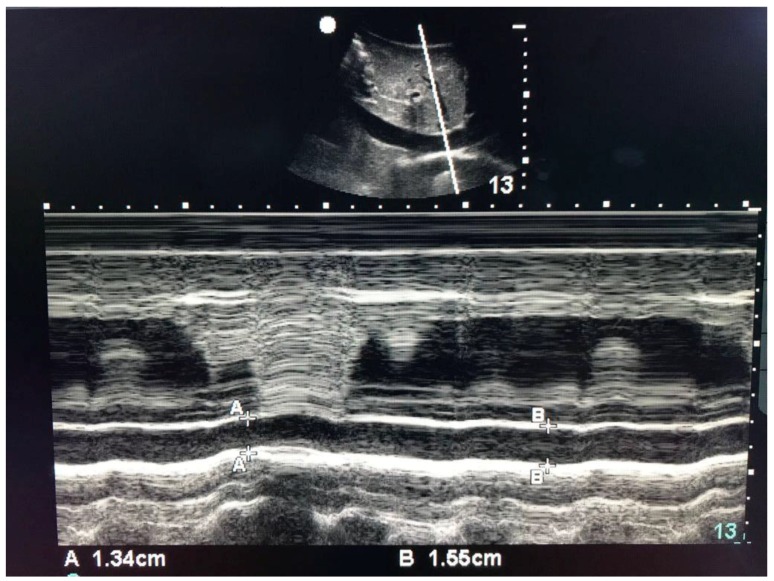
Ultrasound measurements of inferior vena cava (IVC). Panel above shows two-dimensional scan of the IVC with right atrium to the left and panel below shows M-mode scan with respiratory variations in diameter. dIVCmax = maximum diameter of IVC; dIVCmin = minimum diameter of IVC.

USG images of the inferior vena cava of the patients are shown in Figure 2. The two-dimensional scan of the IVC with right atrium to the left are shown in the panel above. The M-mode scan with respiratory variations in diameter are shown in the panel below.

The mean, standard deviation, and minimum and maximum values of the inferior vena cava diameter on inspirium, expirium, and collapsibility index are shown in Table 2. The maximum diameter of the inferior vena cava were 1.99 ± 0.31 mm preoperatively and 2.05 ± 0.29 mm postoperatively (*p* > 0.05). The preoperative IVC-CI was 14.0 ± 9.60% preoperatively and 15.14 ± 11.18% postoperatively. No statistically significant difference was determined between the mean inferior vena cava diameters on inspirium, expirium, and collapsibility index preoperatively and postoperatively (*p* > 0.05). There was no significant difference between hemodynamic parameters (systolic, diastolic, mean blood pressure) of preoperative and postoperative periods (*p* > 0.05).

**Figure 3:**
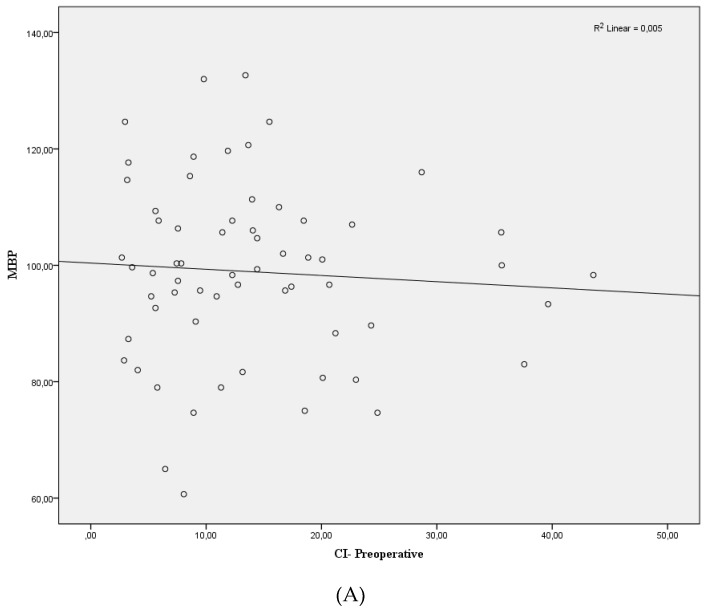

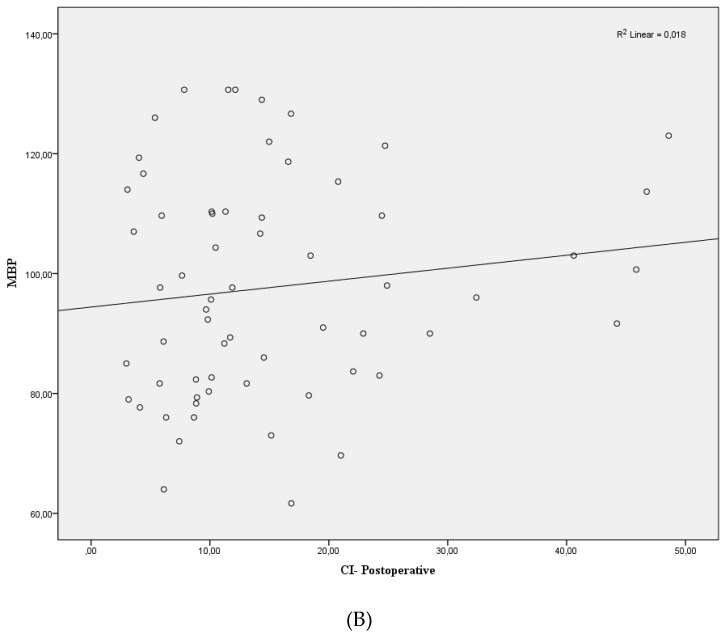
Scatter plots showing the relationships of preoperative (**A**) and postoperative (**B**). Mean blood pressure (MBP) and collapsibility index (CI) of inferior vena cava. *p* > 0.05.

**Figure 4:**
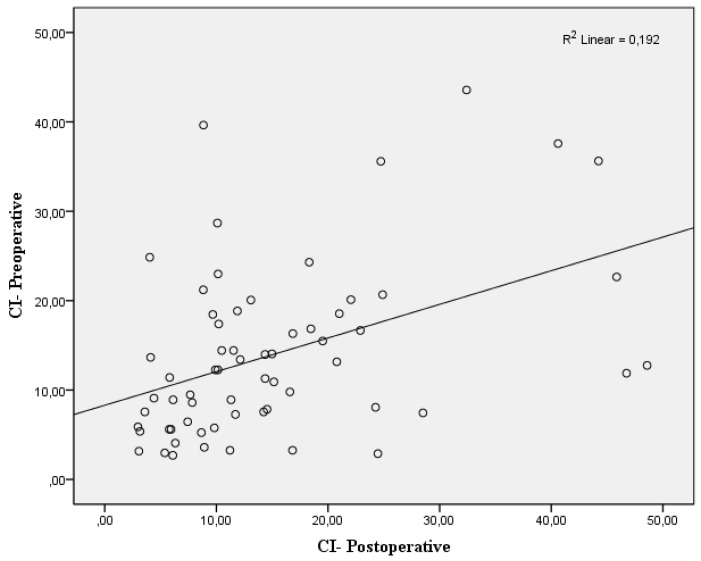
Correlation of the collapsibility index (CI) of inferior vena cava between preoperative and postoperative period. *p* < 0.01.

No significant differences were seen between mean arterial pressure and dIVC postperatively (Figure 3A). The correlation coefficient was determined as *r* = 0.018 (*p* = 0.31). Similar results were determined in the relationship between mean arterial pressure and dIVC preoperatively (*r* = 0.005, *p* > 0.05) (Figure 3B). Positive and statistically significant correlation was found between preoperative CI and postoperative CI (*r* = 0.438, *p* < 0.01) (Figure 4).

## 4. Discussion

In our prospective study, we determined the changes of the inferior vena cava diameter and collapsibility index values preoperatively and postoperatively. We did not find any significant difference of the IVC measurement values between the pre- and postoperative periods in accordance with hemodynamic parameters.

In recent years, bedside ultrasongraphy has gained popularity in anethesiology as being cost-effective, noninvasive, and a practical diagnostic tool, while supplying real-time images. There was much research investigating intravascular volume status by USG of IVC diameters on healthy volunteers [10], peritoneal dialysis patients [11], healthy volunteers in the center of phlebotomy [12], and critically ill patients in intensive care units [13,14] and it was shown to be a safe and reliable method in metanalyses for fluid resuscitation [15].

The variation of IVC diameter is related to compliance of IVC vessel, central venous pressure, and intrathoracic and intra-abdominal pressure. Although there were some studies using (Dmax + Dmin)/2 formula for the calculation of the Caval index (CI) [16], we calculated with the (dIVCmax − dIVCmin)/dIVCmax formula which is mostly used [17,18,19]. Ultrasonography of the IVC was measured in multiple locations in different studies and it was shown that the measurement locations affect the CI values [20]. The upstream origin of the suprahepatic vein [14], at the level of the left renal vein [20], and 10 mm distal to the diaphragm [21] were different locations that IVC measured. In our study, we obtained images from the most frequently used measuring location which was within 2–3 cm from the right atrium outlet [8,14,17]. Nine patients (12.5%) were excluded from the study because IVC ultrasonography measurements were not obtained due to excessive gas, large body size, and increased subcutaneous fat texture in the intestine. Our results were consistent with other studies with unsuccesful IVC visualization observed in 11% [17] and 13.5% [16] of patients in different studies.

The postlaparotomy or increased intra-abdominal pressure may affect the IVC diameter, so these patient groups were excluded from our study [22]. In addition, adequate analgesic supplementation may have been needed for comfortable USG images due to restlessness caused by postoperative pain.

The IVC diameter and IVC variability are effective methods for predicting fluid responsiveness both in mechanically ventilated critically ill patients and spontaneous breathing patients. Studies conducted up to now on the cut-off values of IVC parameters did not result in a common result. The cut-off value for IVC-CI fluid responsiveness in mechanically ventilated septic patients was measured and varied between 12–18% [13,14], whereas in spontaneous breathing patients, Müller et al. demonstrated a cut-off value of 40% [18], Airapetian et al. 42% [17]. Muller et al. showed that patients with cIVC above 40% are more likely to respond to fluid challenge, although values below 40% cannot exclude fluid responsiveness in patients with acute circulatory failure [18]. Due to the uncertainty of the cut-off values in spontaneous breathing patients, we have not used a common value that can show fluid responsiveness in our study.

To prevent the risk of pulmonary aspiration, preoperative fasting protocols are applied before planned surgeries. It is assumed that hemodynamic fluctuations intraoperatively and at the anesthetic induction period that decreased blood volume may be caused by hypovolemia and dehydration due to preoperative fasting [2,16,23]. Opposite to this assumption, other studies like that of Müller et al. stated that there is no significant effect of preoperative fasting on hypovolemia [22]. Jacob et al. stated that healthy patients remain normovolemic after preoperative fasting and hypotension developing after anesthetic induction was not due to hypovolemia [24]. There is evidence that the IVC diameter is a reliable indicator of volume status [25]. The greater collapsibility index with small IVC diameter suggested low volume status in the study of Seif et al. [26]. In our study, we did not detect a higher collapsibility index (14.0 ± 9.60%) and smaller IVC diameters preoperatively. Based on these results, we can say that preoperative fasting does not cause volume changes based on IVC diameter measurements.

Fluid balance in the postoperative period can be predicted by changes in body weight, peroperative fluid input, urine output, hemodynamic parameters, CVP, etc., but the results are not accurate and efficient. For example, low urine output does not mean low intravascular blood volume because the surgical stress increases both antidiuretic hormones and sympathetic tone that lowers urine output. Moreover, large amounts of total blood volume loss may maintain blood pressure at normal levels [27]. Other measurement devices like pulmonary artery catheters, which are used mostly in cardiovascular surgeries, are limited due to practical limitations in elective noncardiac surgeries. Moreover, as the study of Marik stated in their systematic review, CVP should not be used to make clinical decisions regarding fluid management because of the poor relationship between CVP and blood volume as well as the inability of CVP/ΔCVP to predict the hemodynamic response to a fluid challenge [28]. Although CI index change was higher in preoperative patients than in postoperative patients, no significant difference was found in our study. It was also observed that the number of patients with elevated CI increased. Possible causes of these nonspecific results include the changes in the compartments of the fluid in the body due to surgical stress, anesthetic agents, and hypothermia [23,29]. Therefore, we think that ultrasonographic IVC measurements are a simple, practical, and effective method for determining volume status in the postoperative early period.

There are several limitations on this study. In order to confirm these findings, the study population should be large. In our study, because of the measurement of different surgical operations, minor and major surgical procedures cause different levels of stress activation and consequently the fluid shifts between compartments may differ and these can cause different measurement results. For this reason, it is important to perform separate studies in similar surgical procedures. In addition, since the perioperative fluid regimes differ, the work should be extended with different fluid regimes. Because the increase in intra-abdominal pressure is a limiting factor for IVC measurements, another measurement for dIVC method should be performed for this group of patients.

## 5. Conclusions

In conclusion, ultrasonographic IVC measurements of postoperative patients and CI calculation were not found to be statistically higher than preoperative patients despite standard peroperative fluid treatments. IVC diameter measurements are an effective, practical, noninvasive method for demonstrating fluid responsiveness that can be used safely in fluid management of pre- and postoperative patients. We did not find any similar studies in the literature review on this issue, so this concept can be a sample for large series studies.

## Figures and Tables

**Table 1:** Demographic data of the patients.

Characteristic	Value
Age (years)	42.88 ± 17.38 (18–86)
Gender (M/F)	32/31
Height (cm)	166.37 ± 9.18 (148–186)
Weight (kg)	71.36 ± 11.76 (53–105)
BMI (kg/m^2^)	25.73 ± 4.07 (17.72–37.78)
Operation duration (min)	78.30 ± 45.45 (20–216)
Peroperatively infused fluid	985.80 ± 484.27 (150–2500)
Preoperative hemodynamic parameters	
Preop Systolic BP	136.40 ± 22.17
Preop Diastolic BP	80.14 ± 13.80
Preop MBP	98.88 ± 15.25
ASA	
ASA I	35
ASA II	22
ASA III	6
Comorbidities	
Hypertension	6
Diabetes mellitus	4
Cardiovascular	2
Respiratory	2
Type of surgery	
General surgery	33
Orthopedia	16
Urology	6
Otolaryngology	4
Neurosurgery	4
Planned Anesthesia	
General	39
Spinal	20
Regional block	4
Surgery	
Emergency	16
Elective	47
Total	63

ASA: American Society of Anesthesiologists; mean ± standard deviation; *n*: Patient number; M: male, F: female; BMI: Body Mass Index; BP: Blood Pressure; MBP: Mean Blood Pressure.

**Table 2:** Characteristics of inferior vena cava diameters and hemodynamic parameters preoperatively and postoperatively.

Characteristic	preop	postop	*p*
IVC max diameter (cm ± SD)	1.99 ± 0.31	2.05 ± 0.29	0.063
IVC min diameter (cm ± SD)	1.72 ± 0.33	1.74 ± 0.32	0.407
CI index %	14.0 ± 9.60	15.14 ± 11.18	0.416
SBP	136.39 ± 22.17	135.19 ± 24.19	0.710
DBP	80.14 ± 13.80	78.93 ± 17.26	0.605
MBP	98.89 ± 15.25	97.68 ± 18.24	0.621
Total	63		

CI = collapsibility index; dIVCmax = maximum diameter of IVC; SBP = systolic blood pressure; DBP = diastolic blood pressure; MBP = mean blood pressure; IVC = inferior vena cava.
